# Emergency Response Using Volunteered Passenger Aircraft Remote Sensing Data: A Case Study on Flood Damage Mapping

**DOI:** 10.3390/s19194163

**Published:** 2019-09-25

**Authors:** Chisheng Wang, Junzhuo Ke, Wenqun Xiu, Kai Ye, Qingquan Li

**Affiliations:** 1 Guangdong Key Laboratory of Urban Informatics, School of Architecture & Urban Planning, Shenzhen University, Shenzhen 518000, China; sherwoodwang88@gmail.com (C.W.); liqq@szu.edu.cn (Q.L.); 2 Key Laboratory of Urban Land Resources Monitoring and Simulation, Ministry of Land and Resources, Shenzhen 518000, China; 3 Shenzhen Urban Public Safety and Technology Institute, Shenzhen, Guangdong, China, Shenzhen 518000, China; xwqszu@163.com (W.X.); yekaiszy@163.com (K.Y.)

**Keywords:** emergency response, volunteered remote sensing, passenger aircraft, flood damage mapping

## Abstract

Current satellite remote sensing data still have some inevitable defects, such as a low observing frequency, high cost and dense cloud cover, which limit the rapid response to ground changes and many potential applications. However, passenger aircraft may be an alternative remote sensing platform in emergency response due to the high revisit rate, dense coverage and low cost. This paper introduces a volunteered passenger aircraft remote sensing method (VPARS) for emergency response. It uses the images captured by the passenger volunteers during flight. Based on computer vision algorithms and geocoding procedures, these images can be processed into a mosaic orthoimage for rapid ground disaster mapping. Notable, due to the relatively low flight latitude, small clouds can be easily removed by stacking multi-angle tilt images in the VPARS method. A case study on the 2019 Guangdong flood monitoring validates these advantages. The frequent aircraft revisit time, intensive flight coverage, multi-angle images and low cost of the VPARS make it a potential way to complement traditional remote sensing methods in emergency response.

## 1. Introduction

As early as 1997, Kuroda proposed a global disaster observation satellite system (GDOS) to observe disasters through remote sensing [1]. After decades of development, modern remote sensing technology has made great progress. From the earliest digital imaging, remote sensing technology has been developed with higher spatial resolution, spectral resolution and time resolution, and have been applied in many disaster monitoring fields. In 2008, China launched a microsatellite environmental and disaster monitoring constellation, including two small satellites. One of them was equipped with an interferometric imaging spectrometer (IFIS), which has a good application prospect in environmental and disaster monitoring [2]. The high-resolution digital elevation models and imaging spectra obtained from modern remote sensing technique are playing a significant role in risk assessment of fires, earthquakes and other disasters [3,4]. The development of interferometric synthetic aperture radar (InSAR) remote sensing provides an effective way for ground data measurement and deformation observation with sub-centimeter accuracy. For flood disasters, the flood and landslide areas can be demarcated for assessment and land environment detection [5]. For forest fires, satellite data can be used to identify and map forest fires and record the frequency of different vegetation types or areas affected [6]. Assisted by geographic information systems, the remote sensing data can be used to predict the factors affecting fire occurrence and understand the dynamic range of fire. Lee used GIS and remote sensing technologies to assess the hazards of the Penang landslide, Malaysia, and determined the location of the landslide within the study area through aerial photos and field surveys [7].

Although great progress has been made in modern remote sensing, large-scale disaster response data acquisition based on satellite and airborne platforms remains difficult [8,9]. The high cost and lack of operational flexibility make satellite remote sensing time-sensitive [10]. To achieve a short revisit time, Planet Labs has used several inexpensive microsatellites to provide a complete image of the earth [11], but it is still unable to respond quickly to floods. On the other hand, because the satellite is high above the ground, cloudy weather during rainy days prevents the camera from taking pictures of the ground through the clouds. However, we find passenger aircraft could be a proper remote sensing platform to overcome these limitations. The civil aviation industry has developed to a high level since the 21st century, with up to 8000 routes and nearly 20,000 flights each moment, covering nearly 200 countries and regions, as well as 1700 airports [12]. Using onboard cameras, video or photos of the ground from takeoff to landing can be easily obtained. Lower flight heights and a dense number of flights make aircraft easier to get usable data from. Numerous attempts have been proposed to use the volunteered geographic information (VGI) for flood damage mapping [13,14,15]. Although the quality of volunteered data is not comparable with professional remote sensing, the development of computer image processing algorithms, such as structure from motion (SfM) [16,17], make it possible to retrieve useful geographic information from them.

In this paper, we propose a novel volunteered remote sensing method using passenger aircraft platforms (VPARS). We firstly studied how to generate standard remote sensing products from the images taken in passenger aircraft. Potential applications on flood disaster observations were investigated by a case study of the 2019 Guangdong flood damage mapping. The case study shows that the VPARS can clearly map the flooded area and overcome the cloud cover limitation to some degree. We concluded that the VPARS method can complement the shortcomings of traditional remote sensing in flood damage mapping and has great potential for other emergency response scenarios.

## 2. Method

In the VPARS method, there are mainly three steps to generate the regular remote sensing products. The first step is the data preparation where the positions and interior orientation of the camera for each photo are initialized. Secondly, the structure from motion processing is implemented to estimate the three-dimensional structures and refine the camera positions and orientation. Finally, based on the obtained camera parameters and three-dimensional ground surface, remote sensing products can be generated by the orthorectification and mosaic of the original photos.

### 2.1. Initialization of Image Positions and Interior Orientation Parameters

**Figure 1:**
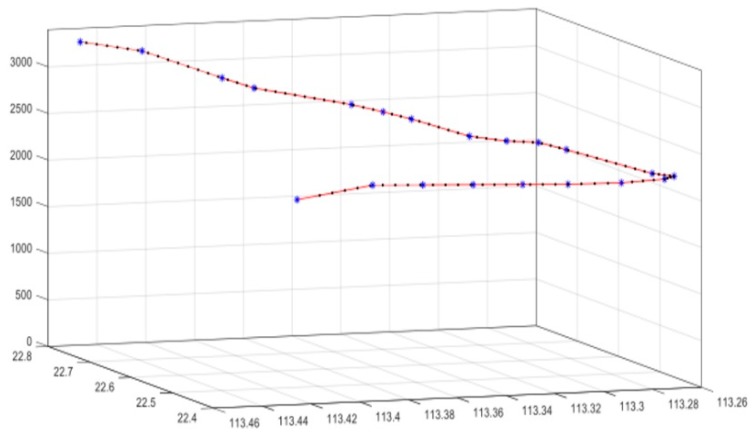
Piecewise linear interpolation of the discrete aircraft positions downloaded from FlightAware. Blue asterisks are the original flight tracking points with position information, and black points are the image locations interpolated from original flight tracking points.

After decades of development, the modern aircraft is equipped with many advanced equipment, including high definition cameras and modern radiocommunication systems. Although cameras on aircraft can clearly capture high-quality images of the ground, these images lack the corresponding coordinate information for matching. Fortunately, the flight line coordinates of modern aircrafts can be acquired from the Aircraft Communication Addressing and Reporting System (ACARS) and Automatic Dependent Surveillance Broadcast (ADS-B), which is collected by commercial companies for flight tracking services, and we can normally obtain the GPS positions of a flight with a certain sampling frequency (e.g., ~1/30 Hz in FlightAware [12]) from this information. As for the stable speed of aircraft, the initial camera position can be calculated by the piecewise linear interpolation method (Figure 1).

To facilitate conjugate point searching, we also need the approximate interior orientation parameters, including the sensor size and focal length. These parameters can be read from the exchangeable image file (EXIF) header of the images. In the next step, the more precise values of the sensor size, focal length and other interior camera orientation parameters will be determined by SfM processing [18].

### 2.2. Structure from Motion Processing

**Figure 2:**
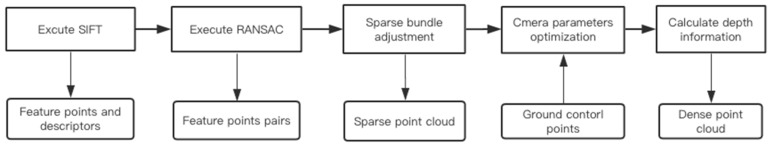
Structure from motion (SfM) workflow.

SfM is a three-dimensional reconstruction method based on unordered images, and many software programs are available for SfM processing, e.g., Pix4d, VisualSFM, Agisoft Photoscan and Menci APS. We adopted Agisoft Photoscan for SfM processing in this work [19]. Before performing core calculations, appropriate images were selected to determine the camera parameters. Considering the algorithms used in this software are not fully described, here we present the SfM workflow as follows (Figure 2):

**Step 1:** Detect features in each image by using the SIFT algorithm [20]. In this step, the Gaussian function was used to convolve the input image. The Gaussian pyramid was obtained after repeatedly applying convolution. By subtracting the adjacent level of the Gaussian pyramid, the difference of Gaussian (DOG) can be obtained. Key locations were then defined as maxima and minima from the result of the DOG function. Low-contrast points were discarded, and the rest of them were located as stable feature points. Next, one or more orientations were calculated for each feature point ($A_{ij}$), where the image gradient magnitude ($M_{ij}$) and orientation ($R_{ij}$) were computed using pixel differences:

(1) $$M_{ij}=\sqrt{{\left(A_{ij}-A_{i+1,j}\right)}^{2}+{\left(A_{ij}-A_{i,j+1}\right)}^{2}},$$

(2) $$R_{ij}=atan2\left(A_{ij}-A_{i+1,j},\;A_{i,j+1}-A_{ij}\right).$$

**Step 2:** Use the kd-tree model to calculate the Euclidean distance between two image feature points for feature point matching and finding an image pair with the required number of the feature points.

**Step 3:** For each pair, estimate an F-matrix and refine matches by using the Random Sample Consensus (RANSAC) algorithm [21]. If feature points can be chained in matching pairs and detected, then a track can be formed.

**Step 4:** Use the sparse bundle adjustment (SAB) to estimate the camera position and extract a low-density point cloud [22,23]. We delivered the feature points from RANSAC to SAB to estimate the camera position and extracted a low-density point cloud. Based on the projection function π, for each point pair, $E_{i}^{j}$ is defined as the difference between the image reprojection ${\tilde{x}}_{i}^{j}$ of map point *j* in the $i^{th}$ image, and its actual image measurement $x_{i}^{j}$:

(3) $$E_{i}^{j}={\tilde{x}}_{i}^{j}-\pi \left(R_{i},T_{i},x_{i}^{j}\right).$$

Define $R_{i}\;\mathrm{and}\;t_{i}$ as the camera pose of all frames and $X_{i}^{j}$ as the corresponding 3D coordinates of each feature point, such that the bundle adjustment optimization model can be written as Equation (4). Here, for each feature point, the residual error can be represented by $E_{i}^{j}$, and $\Omega_{i}^{j}$ is the information matrix of the feature point and is used for weight representation. During the bundle adjustment processing, the poses of all frames and the 3D information of the feature points are optimized, except for the original frames that are fixed to eliminate the gauge freedom.

(4) $$F\left({\left\{R_{i},t_{i}\right\}}_{i=1..m},{\left\{X_{i}^{j}\right\}}_{j=1..N}\right)=\overset{m}{\underset{i=1}{\sum }}\overset{N}{\underset{j=1}{\sum }}{\left(E_{i}^{j}\right)}^{T}\Omega_{i}^{j}\left(E_{i}^{j}\right).$$

Thus, the bundle adjustment optimization procedure means solving the following minimizations problem:

(5) $$\left({\left\{R_{i},t_{i}\right\}}_{i=1..m},{\left\{X_{i}^{j}\right\}}_{j=1..N}\right)\underset{R_{i},t_{i},X_{i}^{j}}{=\mathrm{argmin}}\;F\left(R,t,X\right).$$

This can be solved by iterations of reweighted nonlinear least squares. One fundamental requirement to do this efficiently is to differentiate measurement errors and to obtain their Jacobians with respect to those parameters that need to be estimated.

**Step 5:** Optimize the internal and external camera orientation parameters using ground control points (GCPs).

**Step 6:** Calculate depth information for each camera and combine them into a single dense point cloud.

### 2.3. Orthoimage Mosaic Generation

**Figure 3:**
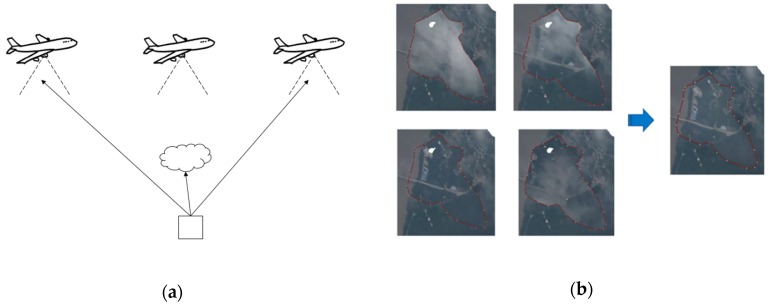
(**a**) A sketch map showing different viewing angles during flights, and (**b**) an example showing how multi-view images generate cloud-free images.

When the SfM workflow is finished, a dense point cloud can be obtained. At the same time, a digital surface model (DSM) in the form of raster image can be generated from the regular interpolation of the point cloud. With the DSM and optimized camera positions and orientation parameters, the multiple input photos are orthorectified to orthophotos. To mosaic the orthophotos, several strategies are practicable such as averaging the values of all pixels from individual photos, taking pixels from the photo observations closest to the normal direction or using a frequency domain approach [19]. More importantly, a notable feature of the VPARS is that the height is significantly lower than satellites. When the aircraft pass through the cloud, the photos captured in different positions have different blind areas blocked by clouds (Figure 3a). Therefore, it is possible to generate cloud-free orthoimage by combining these multi-view photos. The principle is similar to the previous cloud-free generation methods using time-series satellite images with different cloud cover [24,25]. In this case, to remove cloud cover in the mosaic photo, we manually chose the images in the cloud cover area for generating the orthomosaic image. Thus, the final orthomosaic can be free of cloud according to our selection (Figure 3b).

## 3. Results

### 3.1. Study Area

In June 2019, heavy rain and floods attacked southern China’s Guangdong province, and destroyed roads and toppled houses. According to the local publicity department, 13 people were killed and two were still missing. More than 280,000 people were affected and over 4000 houses collapsed [26]. The rapid mapping of the flood area is extremely important for local authorities, who rely on such information to determine the number of quilts, tents, folding beds and other disaster relief to be supplied to the affected area.

Guangdong is the most populous province of China and the 15th-largest by area, a coastal province in South China on the north shore of the South China Sea. It has a humid subtropical climate and routinely suffers from droughts. However, its economy is larger than that of any other province in the nation and the 6th largest sub-national economy in the world in 2018. The flood events may therefore cause severe damage to this area. The VPARS method is a suitable way for flood damage mapping in the Guangdong Province. The developed air traffic over there allows dense observation and high observation frequency. Moreover, the cloud-free map generation capability of VPARS by combining multi-view photos can overcome the frequent cloud cover problem in southern China.

In this case, we processed a series of photos taken by a passenger volunteer on flight CZ6591, from Shenzhen to Ningbo, on June 25, 2019 (09:35 am, local time). These photos were taken over a part of Dong River, from Taimei town to Guanyinge town in Huizhou City, across a length of about 16.42 km. Dong River is the eastern tributary of the Pearl River in the Guangdong province, southern China. It is one of three main sources of floods in the Pearl River Delta, the other two are Xi River and Bei River [27]. The biggest flood peaks in Dong River mostly occur from May to June, lasting 7 to 15 days [28]. In history, there were 14 major floods in the Dong River Basin from 1864 to 1985. The Dong River and its tributaries are also the source for the June 2019 Guangdong flood. This observation provides an opportunity to validate the capability of the VPARS to complement traditional satellite remote sensing for rapid flood damage mapping.

### 3.2. Data Processing

We downloaded the flight track log from the FlightAware website to obtain coarse GPS trajectory positions of flight CZ6591 every 30 s. Based on the timestamp of each image, we ran a piecewise linear interpolation on the discrete flight position data to obtain the camera position for each image. Considering the error between reported flight position to interpolation, the measurement accuracy for the camera position was set to 100 m.

Then we defined an average ground altitude of 0 m to avoid computation errors due to the very oblique shooting angle. The focal length was preset as 4 mm and sensor size as 2.65 mm × 1.99 mm as read from the image head file captured by the camera. The camera position and extract sparse point clouds were further estimated by applying sparse bundle adjustment.

**Figure 4:**
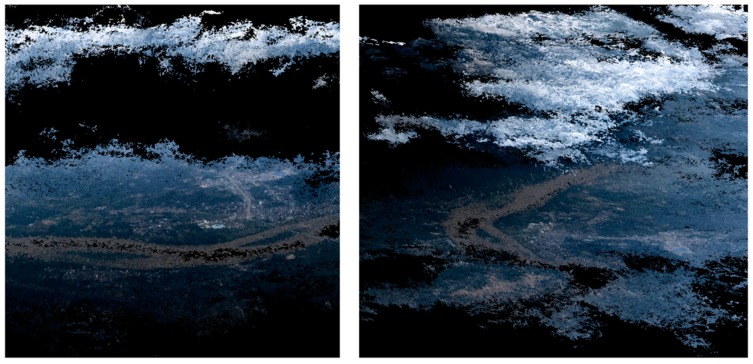
The derived point cloud shows that the clouds above the ground are clearly identified.

A preliminary orthostatic image was derived based on the sparse cloud points. To locate the ground control points (GCPs), we used Google Earth images as reference. The heights of these GCPs were acquired from the Advanced Spaceborne Thermal Emission and Reflection Radiometer (ASTER) global digital elevation model (GDEM). In this paper, to be consistent with the GDEM height, these GCPs are all selected on the ground. None of them were on surface objects like buildings. With the GCPs and preliminary camera positions, we used the SfM step mentioned before to generate the dense cloud point. The cloud point is filtered firstly by the Local Outlier Factor (LOF) method [29], and some remaining noisy points are further removed manually. The clouds above the ground are clearly identified as shown in Figure 4. To remove the cloud effect on the orthomosaic image, we interactively chose the images in which we could observe the ground as the source for orthomosaic image generation in the cloud-cover area.

### 3.3. Results

**Figure 5:**
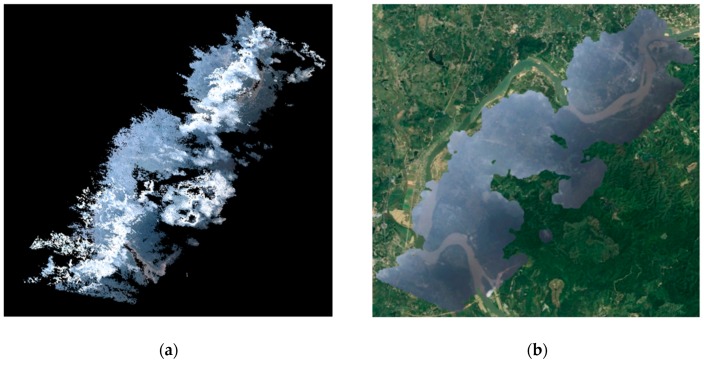
(**a**) Point cloud data, and (**b**) an orthomosaic image of the study area.

The final VPARS products include a dense point cloud and an orthomosaic image as shown in Figure 5. We can see the cloud above the ground is clearly removed in the orthomosaic image, significantly increasing the monitoring area compared to conventional satellite remote sensing. Although the ground sampling resolution cannot compete with UAV images or very high-resolution satellite images, it is still sufficient to distinguish objects at the meter-level, such as roads, buildings and rivers, which is sufficient for flood mapping.

Due to the development of imaging devices, the camera carried by a smartphone can now obtain a relatively high-resolution image for ground observation mission. In this study, the iPhone X dual back cameras used had a 4032 × 3024 image capturing resolution. Given the image data is from the volunteer company or individual, data which cannot be requested from a professional perspective, we used the default setting of the camera during the flight. However, it is possible to avoid the impact of sunlight by previously selecting the opposite seat from the sunlight and taking a perpendicular shooting angle to the ground as much as possible without internal objects from the aircraft. In this case, we took 40 images as data and the exposure time was 1/750 s. The ground sampling resolution (GSR) of orthomosaic image was 1.17 m/pix, calculated by

(6) $$\mathrm{GSR}=\frac{flying\;distance\times physical\;dimension\;of\;sensor}{lens\;focal\;length\times pixel\;dimension\;of\;image}.$$

**Figure 6:**
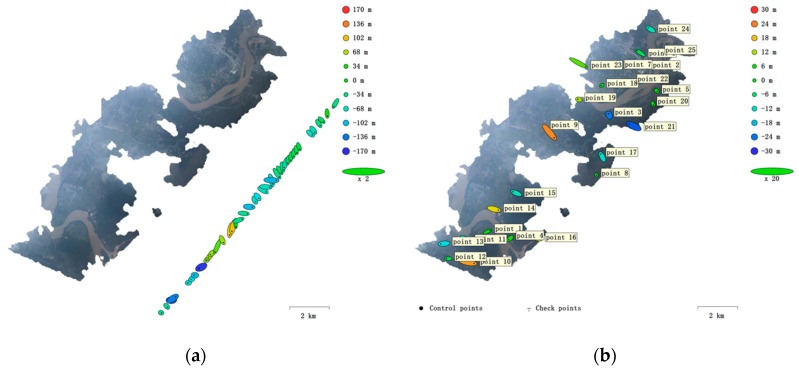
(**a**) Camera locations and error estimates, and (**b**) ground control points (GCP) locations and error estimates. Z error is represented by ellipse color. X, Y errors are represented by ellipse shape.

However, we should note that the image from the camera has a shooting angle and the flying height cannot represent the flying distance directly, so the effective resolution should be larger than the calculated GSR. In this case, according the quality of the initial orthomosaic image using the default resolution, we found the effective resolution should be around 5 m/pix, so we reset the pixel size as 5 m for the final orthomosaic image exportation. We also estimated the camera position error and GCPs error in this study. As shown in Figure 6, the camera position error of 40 images reaches 172.38 m (this includes an estimation error of 95.96 m for longitude, 123.21 m for latitude and 72.97 m for altitude). The GCPs horizontal error is estimated to be 17.73 m. Apart from the time consumed in manual operations, the whole processing time of the algorithms was only about 5 min, most of which is spent on dense point cloud generation and orthomosaic processing. The low time-cost implies the VPARS is capable for fast emergency response and large-scale area monitoring.

**Figure 7:**
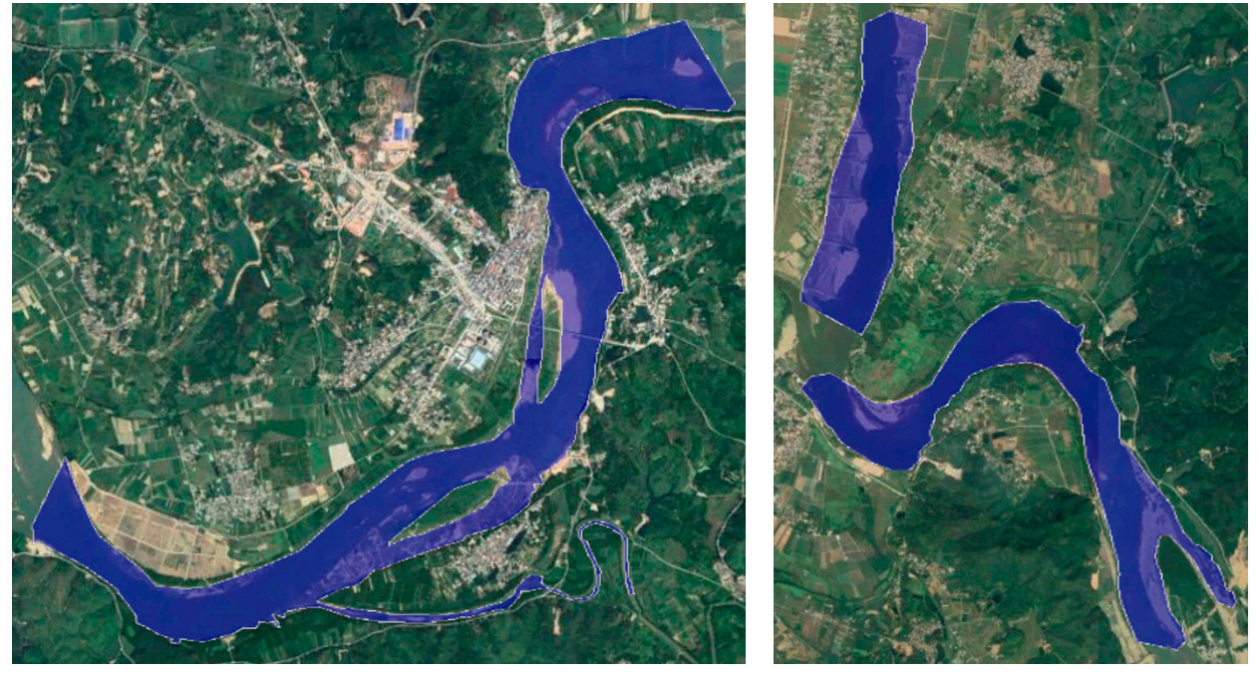
Flood map overlaid on a Google Earth image.

In the scenario of flood monitoring, remote sensing technology can be used to detect the location and scope of disasters, analyze water volume and depth, and assess the impact of floods on cultivated land, residential areas, towns and roads. In this case, the results of the VPARS image can also be applied to identify and determine the flood range. As shown in Figure 5b, the flooded area can be clearly visible in the VPARS orthomosaic image. To have a more precise result, we used a manual way to extract the flooded area along the Dong river in this study. The damage map was then overlaid on Google Earth image as shown in Figure 7. We can clearly observe many cropland, roads, buildings, and grassland were destroyed in this flood.

**Figure 8:**
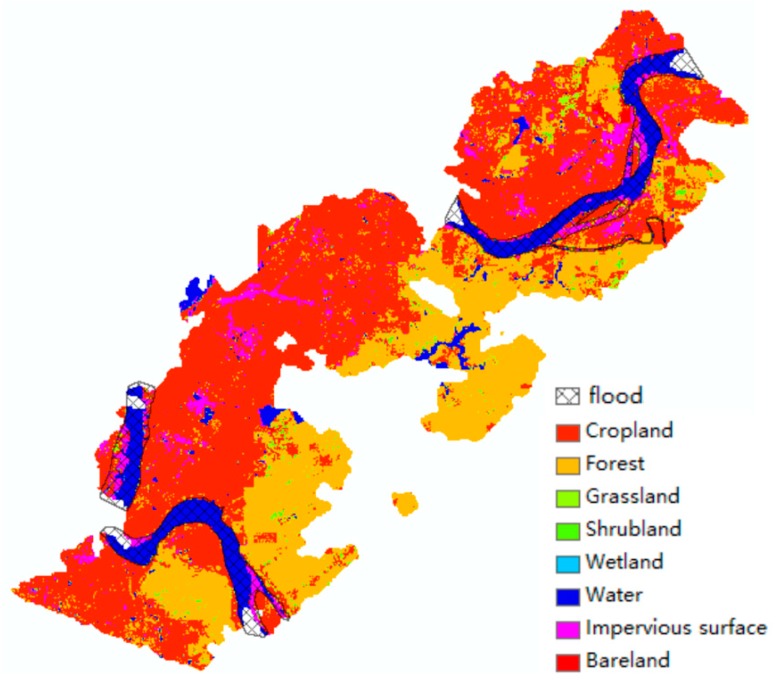
Flood map overlaid on the FROM-GLC 2017 land cover map.

As the Google Earth image does not contain classification information of ground objects, we further overlaid the flood damage map to a FROM-GLC map for more detailed analysis of flood detection results (Figure 8). The full name of FROM-GLC is Finer Resolution Observation and Monitoring of Global Land Cover, which is obtained by Gong et al. with a 10 m resolution [30,31]. The areas affected by flood and waterlogging can be calculated by a simple GIS spatial analysis step. We found the largest area where the flood impacted was 0.965 km^2^ in the impervious surface surrounding the river, implying significant influence of this flood to areas of human activity. The second affected ground object was the cropland, which also suffers greatly from floods and a 0.74 km^2^ affected area was detected. Other affected ground objects vary from 0.003 to 0.13 km^2^, details of which are shown in Table 1.

### 3.4. Validation with Sentinel-1 SAR Image

To validate the flood mapping results, we used the conventional satellite remote sensing data for comparison. As the cloud coverage is high around 25 June 2019 over the study area, there is no cloud-free optical remote sensing data available from some typical satellites such as Planet, Sentinel-2 and Landsat. However, SAR (Synthetic Aperture Radar) can penetrate cloud cover and is sensitive to water, which is widely applied in flood mapping. Here, we used a Sentinel-1A SAR L1 Ground Range Detected product (10 m resolution in IW mode) to retrieve the flood damage map for comparison. The SAR image was captured in an ascending orbit (No. 27835) with VV polarization on June 25, 2019 (18:25 am, local time), just 9 h after the VPARS observing time in this study. Some quantitative metrics were used to evaluate, defined as follows:

(7) $$\mathrm{Recall}=\frac{tp}{tp+fp},\;\mathrm{Precison}=\frac{tp}{tp+fn},\;F-\mathrm{score}=\frac{2\times \mathrm{Precison}\times \mathrm{Recall}}{\mathrm{Precison}+\mathrm{Recall}},$$

where tp, fp, tn and fn represent the true positive, false positive, true negative and false negative in the confusion matrix, respectively.

**Figure 9:**
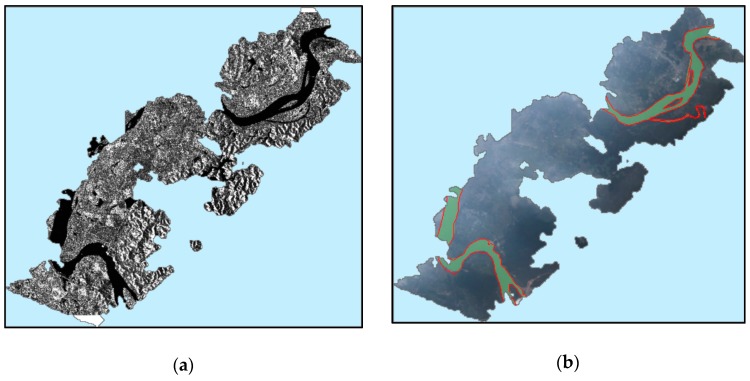
(**a**) Sentinel-1A SAR image. (**b**) Flood map retrieved from SAR (red lines) and volunteered passenger aircraft remote sensing (VPARS) images (green polygons).

**Figure 10:**
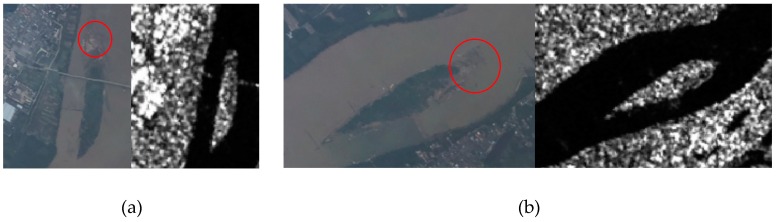
Typical discrepancies between flooded areas detected by (**a**) VPARS and (**b**) Sentinel-1 SAR images.

Due to the low backscatter from the water body, the flooded area is clearly characterized by a dark color (Figure 9a). We also drew the flooded area manually through visual interpretation. The visual interpretation is not time-consuming for this relatively small study area but can give a robust estimation. The flood map from the SAR image agrees well with our VPARS results (Figure 9b). The quantitative assessment gives a high F-score of 0.9308. The recall and the precision are given as 0.9241 and 0.9377, respectively (Table 2). The main discrepancies between two flooding maps are located in the two islands in the river (Figure 10). The VPARS image suggests a less flooded area than SAR. Two possible reasons may explain these discrepancies. First, the flooded area may expand in the 9 h between the VPARS and SAR capturing time. Second, the half-flooded bare soil close to the flooded area also has a very low backscatter value similar to the water body. Considering there is no significant flood expansion in other regions, it is more likely that the SAR image cannot separate half-flooded soil from the water body.

## 4. Discussion

### 4.1. Rapid Response to Emergency

As flood is a natural disaster with short-lived and large-scale impacts, remote sensing should provide reliable and timely damage assessment. To evaluate the response speed of satellite remote sensing, we investigated the data availability from Planet, which is the world’s first company to revisit any location in a single day by using a fleet of Dove satellites to provide global observations for many customers around the world with 3–5 m resolution. We set the search area the same as the observing area of our study, which is located from Taimei town to Guanyinge town in Huizhou City, in the Guangdong province. Although Planet can achieve the shortest revisit period among all commercial satellite plans, our searching results in this region show the Planet data were still not available for eight days in June, 2019. In addition, Planet’s coverage in the region is not stable, only 11 days have area coverage larger than 50% (Table 3). Landsat and COSMO-SkyMed 4 are also popular data sources for assessing inundation extent based on multispectral and microwave imaging. Landsat 8 is an equipped operational land imager, which provides 30 m spatial resolution multispectral data, and the revisit time is 16 days. The spatial resolution of COSMO-SkyMed 4 is 5 m with 5 days revisit time. Their long revisit periods make it difficult to respond to flood rapidly.

**Figure 11:**
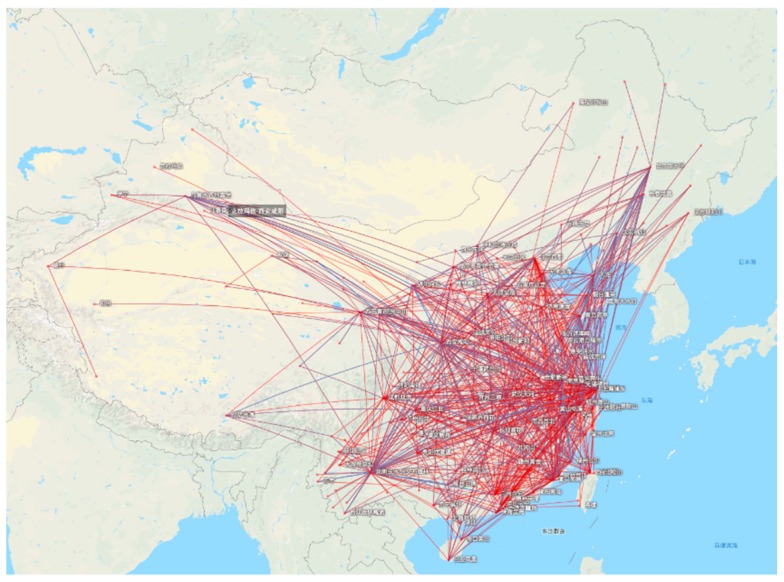
Flight lines in China on June 25, 2019 (https://www.flightaware.com).

However, as shown in the statistics of Guangdong airport routes from Variflight (Table 4), we found that revisiting time and coverage of VPARS is good enough to obtain much more sufficient data than satellite. Because of the high revisiting rate and more flights, it is easier to monitor the regions within the flight route based on the VPARS method [18]. Currently, the satellite can hardly repeat high-frequency surveillance of an area in a few days (or one day), and cannot provide the spatio-temporal coverage required for flood assessment. As flood disaster change continuously in time, disaster management requires up-to-date and accurate flood damage information. Due to the large number of flights and wide-coverage flight lines (Figure 11), the VPARS therefore has the capability to produce timely and accurate flood assessment, which is critically important to prioritize relief efforts and plan mitigation measures against damage.

### 4.2. Cloud-Free Image Generation

**Figure 12:**
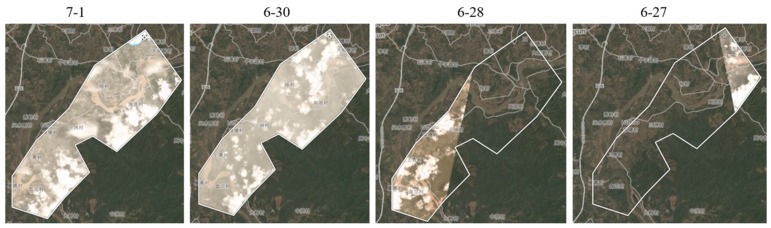
Image availability searched from Planet website (https://www.planet.com/).

Clouds are an important factor preventing remote sensing applications. Normally, clouds are distributed at an altitude of about 2000–6000 m from the ground. And the satellites in remote sensing are usually at an altitude of 400 km above the ground, which makes it easy for them to miss ground images because of cloud cover. We took the remote sensing data from Planet as an example. They are captured by the Dove satellites with orbital heights of 420–475 km. Unfortunately, the Dove satellites did not catch the images covering the experiment area on June 25. Then we searched the Planet images from the surrounding days in June as comparison (Figure 12). At this altitude, the Dove satellites cannot see the ground data directly through the clouds when it takes images of the ground. As shown in the searching results (Figure 12), all these images are covered by clouds, some of them are even incomplete. It is therefore very challenging for satellite remote sensing to overcome the cloud cover limitation.

**Figure 13:**
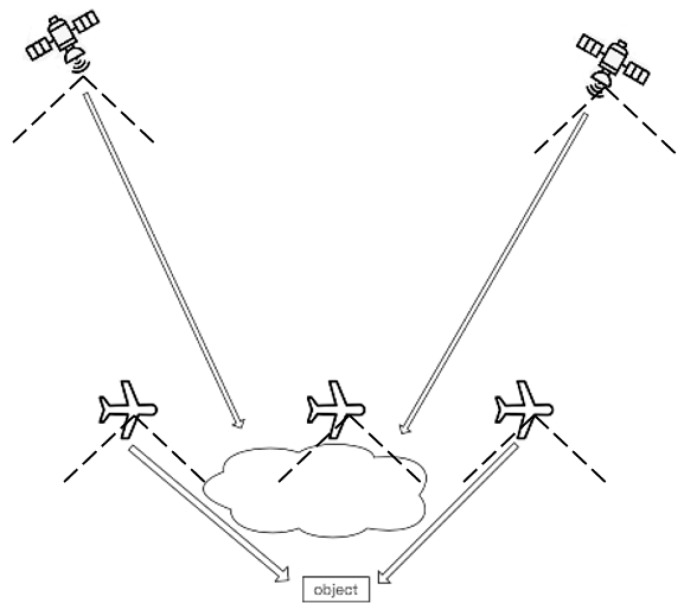
A sketch map showing cloud impact difference on satellite and aircraft.

In the VPARS, since the flight altitude of the aircraft is generally between 6000 and 12,000 m, the relatively lower observation height makes it easier for aircraft camera to break through the cloud interference and obtain the ground data as shown in Figure 13. However, it cannot eliminate cloud cover entirely in a single photo. So, in our study, we used a stacking approach to combine cloud-free part in several images from different angles (Figure 3b). The cloud influence can therefore be easily removed in the VPARS.

### 4.3. Some Limitations

Although the VPARS has the advantages of rapid response and cloud removal for flood disaster management, there are still several limitations to this method. The first shortage we find is image quality. VPARS data is captured by customer-level cameras from passenger volunteers, and the vibration during the flight and shooting the photo through the thick glass from aircraft cabin windows may produce images with low quality, which may aggravate image processing. Conversely, professional optical RS cameras for satellite remote sensing can provide a series of stable, high-precision and easy-to-handle images. Another drawback is the geometric accuracy. As there is no high-precision position and orientation system in customer-level cameras, the geometric error is relatively larger than high-resolution optical satellite remote sensing products. The detailed comparison between VPARS and satellite RS are listed in Table 5.

## 5. Conclusions

In summary, we have proposed a novel remote sensing method using a passenger aircraft platform for flood observation. Currently, flood remote sensing is mainly limited by satellite orbit revisit time and cloud cover, resulting in delayed, partial coverage and incomplete flood assessments. However, a timely and accurate flood assessment is critically important for flood mitigation and response. The proposed VPARS method has advantages, such as a high-revisit period because of the frequent passenger aircraft flights, and the relatively low flight altitude of passenger aircrafts makes it possible to observe ground objects underneath clouds. The cloud-cover influence can therefore be removed by combining multi-view images taken in the passenger aircraft. This case study has validated the capability of the VPARS in flood-damage mapping and cloud-free orthomosaic image generation. However, VPARS also faces challenges in data quality and geometric accuracy. In future work, efforts can be made in fusing multi-source high-quality satellite images and developing information retrieval methods [32] to improve the VPARS data quality.

## Figures and Tables

**Table 1:** Statistics of flooded area.

Ground Object	Area (km^2^)
Cropland	0.7377
Forest	0.0613
Grassland	0.1334
Shrubland	0.0032
Wetland	0.0350
Impervious surface	0.9650
Bareland	0.0843

**Table 2:** Statistics of VPARS flood mapping validation.

Metrics	Value
Recall	0.9241
Precision	0.9377
F-score	0.9308

**Table 3:** Area coverage from Planet in June.

Date	Area Coverage (%)									
June 1–June 10	5	6	0	35	20	26	57	77	79	8
June 11–Jun 20	0	0	0	37	0	63	64	40	50	80
June 21–Jun 30	60	66	0	0	64	0	63	39	60	60

**Table 4:** Statistics of Guangdong Airport Routes on June 25, 2019.

	CAN	MXZ	FUO	SWA	LDG	SZX	HUZ	ZUH	ZHA	HSC
Connection cities	179	6	9	45	0	138	31	56	34	0
Flight routes	422	9	24	106	0	377	65	163	73	0
Flights	1364	13	24	167	0	1036	78	399	96	0

**Table 5:** Comparison between passenger aircraft remote sensing (RS) and satellite optical RS.

	Rapid Response	Less Limited by Cloud	High Image Quality	High Geometric Accuracy
Passenger aircraft RS	Yes	Yes	No	No
Satellite optical RS	No	No	Yes	Yes

## References

[1] Kuroda T., Koizumi S., Orii T. (1997). A Plan for a Global Disaster Observation Satellite System (GDOS).

[2] Wang J., Sammis T.W., Gutschick V.P. A remote sensing model estimating lake evaporation. Proceedings of the IEEE International Geoscience & Remote Sensing Symposium.

[3] Wang C., Li Q., Liu Y., Wu G., Liu P., Ding X. (2015). A comparison of waveform processing algorithms for single-wavelength LiDAR bathymetry. ISPRS J. Photogramm. Remote Sens..

[4] Wang C., Ding X., Shan X., Zhang L., Jiang M. (2012). Slip distribution of the 2011 Tohoku earthquake derived from joint inversion of GPS, InSAR and seafloor GPS/acoustic measurements. J. Asian Earth Sci..

[5] Tralli D.M., Blom R.G., Zlotnicki V., Donnellan A., Evans D.L. (2005). Satellite remote sensing of earthquake, volcano, flood, landslide and coastal inundation hazards. ISPRS J. Photogramm. Remote Sens..

[6] Jaiswal R.K., Mukherjee S., Raju K.D., Saxena R. (2003). Forest fire risk zone mapping from satellite imagery and GIS. Int. J. Appl. Earth Obs. Geoinf..

[7] Lee S. (2005). Application of logistic regression model and its validation for landslide susceptibility mapping using GIS and remote sensing data. Int. J. Remote Sens..

[8] Boccardo P., Tonolo F.G. (2015). Remote sensing role in emergency mapping for disaster response. Engineering Geology for Society and Territory-Volume 5.

[9] Voigt S., Giulio-Tonolo F., Lyons J., Kučera J., Jones B., Schneiderhan T., Platzeck G., Kaku K., Hazarika M.K., Czaran L. (2016). Global trends in satellite-based emergency mapping. Science.

[10] Whitehead K., Hugenholtz C.H. (2014). Remote sensing of the environment with small unmanned aircraft systems (UASs), part 1: A review of progress and challenges. J. Unmanned Veh. Syst..

[11] Boshuizen C., Mason J., Klupar P., Spanhake S. Results from the planet labs flock constellation. Proceedings of the AIAA/USU Conference on Small Satellites.

[12] (2018). FlightAware. https://flightaware.com/.

[13] Rosser J.F., Leibovici D.G., Jackson M.J. (2017). Rapid flood inundation mapping using social media, remote sensing and topographic data. Nat. Hazards.

[14] Schnebele E., Cervone G. (2013). Improving remote sensing flood assessment using volunteered geographical data. Nat. Hazards Earth Syst. Sci..

[15] Poser K., Dransch D. (2010). Volunteered geographic information for disaster management with application to rapid flood damage estimation. Geomatica.

[16] Snavely N., Seitz S.M., Szeliski R. (2008). Modeling the World from Internet Photo Collections. Int. J. Comput. Vis..

[17] Westoby M., Brasington J., Glasser N.F., Hambrey M.J., Reynolds J.M. (2012). Structure-from-Motion photogrammetry: A novel, low-cost tool for geomorphological applications. Geomorphology.

[18] Abrams M., Bailey B., Tsu H., Hato M. (2010). The ASTER Global DEM. Photogramm. Eng. Remote Sens..

[19] Agisoft (2018). User Manuals Agisoft PhotoScan Professional Edition.

[20] Lowe D.G. Object recognition from local scale-invariant features. Proceedings of the Seventh IEEE International Conference on Computer Vision.

[21] Fischler M.A., Bolles R.C. (1987). Random Sample Consensus: A Paradigm for Model Fitting with Applications to Image Analysis and Automated Cartography. Read. Comput. Vis..

[22] Lourakis M.I.A., Argyros A.A. (2009). SBA: A software package for generic sparse bundle adjustment. ACM Trans. Math. Softw..

[23] Tang S., Chen W., Wang W., Li X., Darwish W., Li W., Huang Z., Hu H., Guo R. (2018). Geometric Integration of Hybrid Correspondences for RGB-D Unidirectional Tracking. Sensors.

[24] Helmer E.H., Ruefenacht B. (2005). Cloud-Free Satellite Image Mosaics with Regression Trees and Histogram Matching. Photogramm. Eng. Remote Sens..

[25] Min L.I., Liew S.C., Kwoh L.K. Automated production of cloud-free and cloud shadow-free image mosaics from cloudy satellite imagery. Proceedings of the XXth ISPRS Congress.

[26] CHINADAILY Heavy Rain Leaves 13 Dead, 2 Missing in Guangdong. http://www.chinadaily.com.cn/a/201906/13/WS5d0231a7a3103dbf143280a0.html.

[27] Zhang Q., Zhang W., Chen Y.D., Jiang T. (2011). Flood, drought and typhoon disasters during the last half-century in the Guangdong province, China. Nat. Hazards.

[28] Zhen G., Li Y., Tong Y., Yang L., Zhu Y., Zhang W. (2016). Temporal variation and regional transfer of heavy metals in the Pearl (Zhujiang) River, China. Environ. Sci. Pollut. Res. Int..

[29] Wang C., Shu Q., Wang X., Guo B., Liu P., Li Q. (2019). A random forest classifier based on pixel comparison features for urban LiDAR data. ISPRS J. Photogramm. Remote Sens..

[30] Gong P., Liu H., Zhang M., Li C., Wang J., Huang H., Clinton N., Ji L., Li W., Bai Y. (2019). Stable classification with limited sample: Transferring a 30-m resolution sample set collected in 2015 to mapping 10-m resolution global land cover in 2017. Sci. Bull..

[31] Gong P., Li X., Zhang W. (2019). 40-Year (1978–2017) human settlement changes in China reflected by impervious surfaces from satellite remote sensing. Sci. Bull..

[32] Li Y., Zhang Y., Xin H., Hu Z., Ma J. (2017). Large-Scale Remote Sensing Image Retrieval by Deep Hashing Neural Networks. IEEE Trans. Geosci. Remote Sens..

